# Effect of Cold Atmospheric Pressure Argon Plasma Jet Treatment on the Freeze-Dried Mucilage of Chia Seeds (*Salvia hispanica* L.)

**DOI:** 10.3390/foods12081563

**Published:** 2023-04-07

**Authors:** Sebnem Mutlu, Berkay Kopuk, Ibrahim Palabiyik

**Affiliations:** 1 Edirne Food Control Laboratory Directorate, 22100 Edirne, Türkiye; 2 Department of Food Engineering, Faculty of Agriculture, Tekirdag Namik Kemal University, 59030 Tekirdag, Türkiye

**Keywords:** chia mucilage, freeze-drying, cold plasma, rheology, large amplitude oscillatory shear

## Abstract

In the present study, the effects of the treatment of chia seeds with a cold atmospheric pressure plasma jet (CP) using argon as a working gas for different times (30, 60, and 120 s) on the rheological, structural, and microstructural properties of the freeze-dried mucilages at −54 °C were investigated. All mucilage gels showed pseudoplastic flow behavior, and CP treatment of chia seeds increased the viscosity of mucilages, probably due to the cross-linking between polymer molecules. The dynamic rheological analysis revealed that all mucilages were elastic gels and that CP treatment improved the elastic properties in a treatment time-dependent manner. Large amplitude oscillatory shear (LAOS) results showed that freeze-dried mucilages showed Type I strain-thinning behavior. Similar to small amplitude oscillatory shear (SAOS) results, CP treatment has affected and improved the large deformation behavior of mucilages depending on treatment time. Meanwhile, Fourier transform infrared spectroscopy (FTIR) revealed the incorporation of hydroxyl groups onto the surface and the formation of C-O-C glycosidic bonds during plasma treatment. Scanning electron microscope (SEM) micrographs showed the formation of denser structures with CP treatment time. Regarding color properties, CP treatment decreased the lightness values of mucilages. Overall, this study showed that CP is an effective way to modify both the SAOS and LAOS properties of freeze-dried chia mucilage and improve viscosity.

## 1. Introduction

Chia seeds (*Salvia hispanica* L.) are obtained from the chia plant, an annual plant belonging to the Lamiaceae family [1,2]. Chia has gained significant attention in the 20th century, not only due to its high nutritional value but also because of its interesting technological properties. The most promising feature of chia seeds is the fact that they contain 5–6% soluble fiber. These fibers rapidly hydrate when in contact with water, forming a transparent capsule around the seed called mucilage that is used in food products as an ingredient replacer, thickener, stabilizer, etc. [3,4]. 

Since chia mucilage contains other nutritionally important and functional components, such as protein and oil, as well as carbohydrates, it has the potential to replace or substitute commercial thickeners in certain proportions. It has been reported that the mucilage yield varied in the range of 5–15% of the seed weight, depending on the extraction and hydration conditions, such as temperature, time, water/seed ratio, and pH [4,5,6]. Moreover, the procedure used in the drying of mucilage also has a meaningful impact on the technological properties of the obtained product. For example, according to the work of Tavares et al. [7], mucilage obtained through heat treatment showed a more brittle and less uniform microstructure compared to freeze-drying. Moreover, the authors found that the mucilage obtained through a cold extraction and freeze-drying method exhibited higher porosity as well as better solubility and rheological properties, probably due to the preservation of the mucilage structure during the cold extraction method. However, there is a need to find alternative and cheap methods to improve the extraction yield and tailor the technological properties of mucilage in order to increase its usability in food products [3]. 

Using the most general definition, plasma can be described as an ionized gas containing neutral particles and an equivalent number of positive ions and negative electrons [8]. Plasma can be produced by applying energy, such as thermal, electrical, or electromagnetic, to a gas, which increases the kinetic energy of the electrons and leads to collision processes, resulting in the formation of electrons, ions, radicals, and radiation [9]. Depending on the production method, plasmas can be classified into two groups, namely high-temperature and low-temperature plasma. However, since high-temperature plasma is inapplicable in food processing, low-temperature or CP is the main focus of the food industry. 

The CP technique has recently gained attention from numerous researchers as a microbial inhibition and modification method. Ions, free radicals, and highly reactive intermediate species induce lipid oxidation in cell membranes and oxidation of proteins and DNA in microbial cells. Additionally, the bombardment of a biopolymer surface with reactive plasma species and UV-visible radiations has been found to induce etching, cross-linking, and modification of functional properties [10]. In this context, the effect of CP treatment on different starches [11,12] and food gums, such as fenugreek galactomannan [13], gum arabic [14,15], and xanthan [16,17], was investigated, and changes in the rheological and physicochemical properties were reported. In general, gases such as air or oxygen are used to oxidize the surface, while noble gases such as argon and helium induce cross-linking and the formation of active sites for further reactions [18]. For example, Zou, Liu, and Eliasson [19] have found that argon atoms excited by high-energy electrons could trigger dehydration and cleavage between the reducing ends of two polymeric chains (C-OH). However, there is no study in the literature regarding the effect of CP treatment on the rheological properties of freeze-dried chia mucilages. In light of these, this study aimed to investigate the effect of CP treatment using argon as the working gas on the rheological (flow behavior, frequency sweep, and large amplitude oscillatory shear), physicochemical (color), structural (FTIR), and microstructural (SEM) properties of mucilages obtained through the cold extraction and freeze-drying method from CP-treated seeds.

## 2. Materials and Methods

### 2.1. Material

Chia seeds (*Salvia hispanica* L.), originating in Uganda, were purchased from local markets in Turkey. The seeds were stored airtight at +5 °C until analysis. All chemicals used in the analyses were obtained from Sigma (St. Louis, MO, USA) and Merck (Darmstadt, Germany).

### 2.2. Application of Cold Atmospheric Pressure Plasma to Chia Seeds 

The plasma treatment of chia seeds was performed by a cold atmospheric pressure plasma jet (CP) system (Plasmatreat GmbH, Steinhagen, Germany). The system consists of a generator (FG 5001) that allows the formation of the plasma at 1 kVA (~1 kW) power, a plasma jet (RD2004), a carrier platform to adjust the distance to the nozzle, a transformer (HTR12), a carbon filter (DAE10), and gas connectors. Argon was used as the working gas. Chia seeds (10 g) were put into a 250 mL beaker, and the distance from the nozzle to the substrate surface was adjusted manually. Since the seeds can scatter due to the plasma pressure coming out of the nozzle, the distance between the nozzle and the seed surface is adjusted to a fixed value of 15 cm so that the seeds do not scatter and overheat. During the treatment, the beaker was manually shaken in order to provide homogeneity. The environment’s temperature and humidity were 20 °C and 40%, respectively. The treatment was conducted for 30, 60, and 120 s, and samples were coded as CP30, CP60, and CP120, respectively.

### 2.3. Mucilage Extraction 

The extraction of mucilage from chia seeds was carried out using the method proposed by Fernandes and Salas-Mellado [20] with slight modifications. Briefly, 10 g of seeds were mixed with distilled water at a ratio of 1:40 in a beaker (500 mL). The beaker was covered with aluminum foil and kept at room temperature for 30 min with constant stirring at 100 rpm. Following the extraction, the mucilage-containing solution was lyophilized at −24 °C for 24 h. Afterward, the samples were dried at −54 °C under 0.030 mbar pressure for 5 days in a lyophilizer (Martin Christ Lyophilizer, Alpha 1–2 LD, Osterode am Harz, Germany). The mucilage in the dried sample was separated from the seed by sieving it through a mesh screen (600 mic.) for 15 min and stored at +4 °C in airtight packages until the analysis.

### 2.4. Rheological Analysis

For rheological measurements, mucilage gels (1% *w*/*v*) were prepared in distilled water. All rheological properties were determined using a controlled stress rheometer (TA Instruments, Discovery HR-2, ABD) equipped with a parallel plate (40 mm diameter). The rheometer was equipped with a Peltier system, and the temperature was kept at 25 °C in all cases. After loading samples (1.0 mL) and descending the probe to the desired level, the samples were allowed to rest for 2 min. All rheological tests were performed in duplicate. 

#### 2.4.1. Flow Behavior 

Flow curves were recorded in a shear range of 1–100 s^−1^ at 25 °C. Shear stress and viscosity were recorded as functions of shear rate. The experimental data were fitted to three empirical models, namely the Power-law (Equation (1)), Herschel-Bulkley (Equation (2)), and Casson (Equation (3)) rheological models, using the Trios software provided with the rheometer.

(1) $$\mathrm{Power}-\mathrm{law}\;\mathrm{model}:\;\sigma =K{\left(\dot{\gamma }\right)}^{n}$$

(2) $$\mathrm{Herschel}-\mathrm{Bulkley}\;\mathrm{model}:\;\sigma -\sigma_{0}=K{\left(\dot{\gamma }\right)}^{n}$$

(3) $$\mathrm{Casson}\;\mathrm{model}:\;\sigma^{0.5}={\left(\sigma_{c}\right)}^{0.5}k_{c}{\left(\dot{\gamma }\right)}^{0.5}$$

where *σ* is shear stress (Pa), *K* is consistency coefficient (Pa.sn), $\dot{\gamma }$ is shear rate (s^−1^), *n* is flow behavior index, *σ*_0_ is yield stress (Pa), *σ*_c_ is Casson yield stress, and *k*_c_ is Casson viscosity (Pa^0.5^ s^0.5^).

#### 2.4.2. Frequency Sweep 

The frequency sweep measurements were conducted within the linear viscoelastic range at a constant strain (1%) and over the range of 1–100 rad s^−1^. The variations of storage modulus (G′) and loss modulus (G″) of the mucilages were determined as a function of frequency. The degree of frequency dependence of the storage modulus was determined by the Power-law model (Equation (4)):

*G′* = *A*𝜔*^B^* (4)

where *G′* is the storage modulus (Pa), *ω* is the oscillation frequency (rad s^−1^), and *A* and *B* are the constants.

#### 2.4.3. LAOS

LAOS measurements were performed using a stress- and temperature-controlled rotational rheometer (MCR 302, Anton Paar, Austria) in strain-controlled mode. Fifty-millimeter sand-blasted parallel plates and sand-blasted cap plates were used to eliminate the slip effect and reduce water evaporation, respectively. The non-linear rheological properties of mucilage gel samples were determined at a constant frequency of 10 rad s^−1^ and temperature of 25 °C over the range of 0.005–500% strain values. The analysis was performed in duplicate. All the non-linear data were obtained through the use of RheoCompassTM software and Option Raw Data provided by Anton PaarTM. Elastic and viscous Lissajous-Bowditch curves were plotted using OriginPro (ver. 2018) by using the normalized stress (σ(t)/σ_max_), normalized strain (γ(t)/γ_max_), and normalized strain rate ($\dot{\gamma }$(t)/$\dot{\gamma }$_max_) values [21].

### 2.5. Color Analysis 

The color properties of mucilage gels (%1 *w*/*w*) were determined by a Konica Minolta CR-400 (Chroma Meter, Tokyo, Japan) colorimeter. The color parameters L* (brightness), a* (±red-green), and b* (±yellow-blue) of the samples were determined with illumination D65 and a 10° standard observer. At least 3 reads were taken from the surface of every sample [22]. Total color difference (∆E) was calculated using Equation (5):

(5) $$\Delta E=\sqrt{{\left(L_{control}-L_{sample}\right)}^{2}+{\left(a_{control}-a_{sample}\right)}^{2}+{\left(b_{control}-b_{sample}\right)}^{2}}$$

### 2.6. FTIR Analysis

The infrared spectra of chia seed mucilages were recorded using an Attenuated Total Reflectance-FTIR (Bruker Vertex 70 ATR-FTIR, UK). The mucilages obtained from the seeds were first hydrated in ultrapure water (0.8% *w*/*w*) for 2 h at 70 °C. Subsequently, 30 g was dried on a petri dish in a conventional oven at 60 °C for 48 h to obtain a film. The dried film was then analyzed by ATR-FTIR in transmittance mode to characterize the presence of specific functional groups. The data were obtained in the range of wave numbers from 4000 to 600 cm^−1^ during 45 scans at a resolution of 4 cm^−1^. The functional groups were determined by evaluating the obtained database with specific wave numbers [23].

### 2.7. SEM

The microstructure of freeze-dried mucilages obtained from chia seeds was investigated using a SEM (Fei, Hillsboro, OR, USA). First, samples were hydrated in ultrapure water overnight. The following day, samples were fixed in modified Karnovsky’s fixative (3% glutaraldehyde, 2% formaldehyde in 0.1 M phosphate buffer, pH 7.2) for at least 8 h. Once dried, samples were mounted on an aluminum stub, sputter coated with approximately 100 nm of gold (BAL-TEC SCD 005 sputter coater), and viewed at an accelerating voltage of 20 kV [23].

### 2.8. Statistical Analysis

IBM’s Statistical Software (SPSS version 21, IBM Corp., Armonk, NY, USA) program was used for statistical analysis. Data obtained were subjected to analysis of variance (ANOVA), and the differences were determined at the 95% confidence interval (*p* < 0.05). Tukey’s multiple comparison test was applied to compare the means of quantitative data.

## 3. Results and Discussion

### 3.1. Steady Shear Rheological Properties

**Figure 1:**
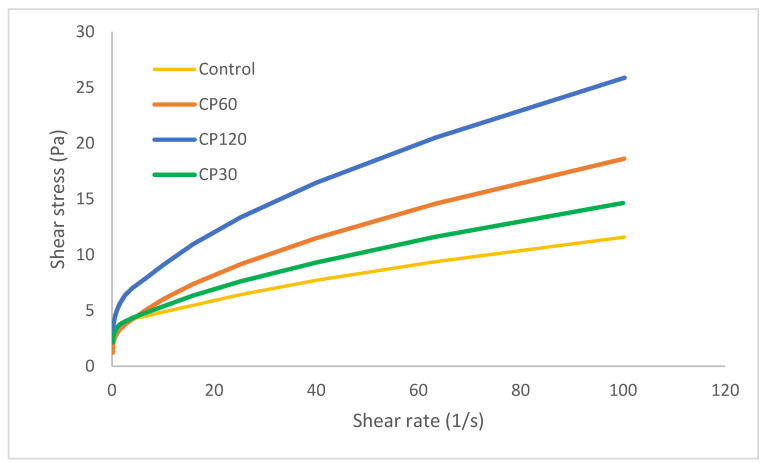
Flow curves of 1% (*w*/*v*) gel of mucilage samples extracted from untreated (control) and CP-treated chia seeds.

Figure 1 shows the flow curves of the chia mucilage samples. As seen, all samples showed non-Newtonian pseudoplastic behavior, where the viscosity values decreased with an increase in the shear rate. Pseudoplasticity is a common behavior for food gums, such as xanthan gum [24], and is also reported for chia mucilage by other researchers [7,25]. This behavior is explained by the orientation effect of polymer chains. At lower shear rates, polymer chains are generally disoriented and only partly aligned, causing high inner friction and viscosity. As the shear rate is increased, the molecules become oriented and aligned in the flow direction, causing lower inner friction and viscosity [26]. 

From Figure 1, it can be said that CP treatment affected the viscosity values of chia mucilages and that there was an increase in the values depending on the treatment time. This may be due to the cross-linking of polysaccharide chains as a result of exposure to highly energetic reactive plasma species. According to Wongsagonsup et al. [27], when the starch is exposed to CP, two competitive reactions, i.e., depolymerization or cross-linking, occur, with one of them dominating depending on the treatment conditions, such as power and time. Moreover, at similar shear rates, the viscosity values of CP-treated mucilage samples were always higher than the control group, with CP120 being the highest. Similar results were also reported for other food gums treated by CP. For example, Misra et al. [16] and Bulbul et al. [17] found that the CP treatment increased the viscosity of xanthan gum solutions due to molecular interactions, such as cross-linking or polymerization of polysaccharide chains. Furthermore, the effect of CP on the viscosity of chia mucilage was more evident at higher shear rates, as reported by Rashid et al. [13] for fenugreek galactomannan. Silva et al. [28] investigated the effects of ultrasound application on the extraction of chia seed mucilage and found that increasing the sonication time decreased the viscosity value at 100 s^−1^. Since the main feature of pseudoplastic fluids is to improve food texture, the higher viscosity values during mastication provide a better mouthfeel upon consumption [26]. Therefore, the CP-treated chia mucilages will provide better textural and rheological properties when incorporated into the food formulations. 

The flow curve data were fitted to the Herschel-Bulkley rheological model using Equation (2), and parameters of the consistency index (*K*), flow behavior index (*n*), yield stress (*σ*_0_), and correlation coefficients (*R*^2^) were given in Table 1. The Herschel-Bulkley rheological model was better at defining the rheological parameters of chia mucilages among other rheological models, i.e., Power-law and Casson (Figure S1), with *R*^2^ values higher than 0.98. Values of *n* < 1 indicate shear-thinning behavior, and lower values mean more pseudoplasticity [27]. Therefore, it can be concluded that the chia mucilage is a shear-thinning fluid. In general, an increase in the concentration of mucilage is accompanied by a decrease in the *n* values due to the presence of more intermolecular interactions, resulting in higher pseudoplasticity [25]. Since the CP treatment decreased the *n* value of chia mucilages, it can be said that exposure to reactive plasma species promoted intermolecular interactions and increased the pseudoplasticity of chia mucilages. However, the decrease in the *n* value was not treatment time-dependent, and the lowest value was obtained in the CP30 group (*p* < 0.05). Similarly, Silva et al. [28] reported that the *n* value of chia mucilage subjected to ultrasound did not show a clear trend with sonication time. The *K* value indicates the viscous nature of the dispersion system [7]. There was a treatment time-dependent increase in the *K* value of chia mucilages, and the highest value was obtained in the CP120 group (*p* < 0.05), suggesting a more viscous network was formed [29]. Yield stress is the minimum shear stress that must be applied to a material to induce flow [30]. The *σ*_0_ value was also affected by CP treatment. Compared to the control, treatment for 30 and 60 s significantly decreased the *σ*_0_ value (*p* < 0.05), while increasing the treatment time to 120 s increased this value insignificantly (*p* > 0.05). Wang et al. [31] investigated the effect of different heat and ultrasound extraction conditions on the rheological properties of the chia mucilage and reported that the best rheological properties were obtained for the mucilage prepared at moderate conditions (50 °C for 30 and 60 min). Moreover, since the chia mucilage is composed of proteins that could affect the flow behavior, the authors proposed that the molecules of polysaccharide in purified mucilage could have a better interaction, causing a higher viscosity.

### 3.2. Frequency Sweep

**Figure 2:**
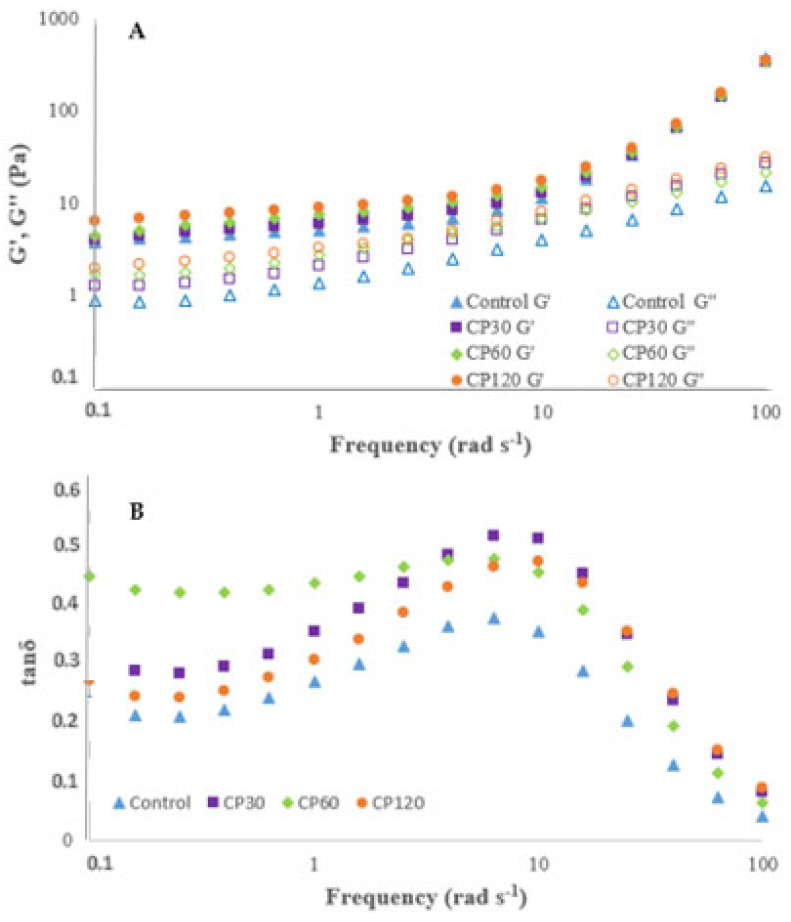
Dynamic mechanical spectrum of gel samples of chia mucilages extracted from untreated (control) and CP-treated chia seeds. (**A**) Storage modulus G′ and loss modulus G″; (**B**) Loss tangent (tan δ).

The viscoelastic behavior of the chia mucilage was investigated by a frequency sweep test. The magnitudes of storage (*G*’) and loss modulus (*G*”) were plotted as a function of angular frequency (*𝜔*) to specify the characteristics of the gel structure in Figure 2. Accordingly, the rheological behavior of all samples was viscoelastic, with *G′* dominating *G*” over the frequency range investigated. Moreover, there was an increase in both moduli parameters with frequency, and no cross-over points were observed, indicating typical weak gel behavior for mucilage samples [32,33]. Goh et al. [23] also found that the gel dispersions of chia seed polysaccharide exhibit weak gel behavior, and they attributed this behavior to the formation of a weak transient gel network connected by the physical contact of neighboring particles caused by the elasticity of the swollen microgel particles dispersed in a soluble polysaccharide fraction. Capitani et al. [25] reported that although there is no cross-over at higher concentrations (0.75% and 1.00%) and the difference between both moduli is greater, the structure of the chia mucilage at lower concentrations (0.25% and 0.50%) broke down at higher frequencies and a cross-over point has been observed. However, for mucilages treated with CP, there was an increase in the moduli parameters depending on the treatment time, with the highest value being recorded for the CP120 group. Similarly, Sadeghi et al. [33] found that exposure to CP treatment at a voltage of 5 kV increased the *G*′ and *G*’’ values of dispersions of *Lepidium perfoliatum* seed gum depending on the treatment time, and they attributed this increment to the presence of more molecular interaction reinforcing the viscoelastic behavior in the treated gum dispersions. Cervantes-Martínez et al. [34] reported that an increase in the concentration of aloe vera mucilage improved the viscoelastic properties due to the presence of more linked points between the polysaccharide chains, causing a greater number of intermolecular interactions. Therefore, it can be concluded that the CP treatment of chia seeds improved the viscoelastic behavior of freeze-dried mucilages due to the formation of more intermolecular linkage points, and this phenomenon intensified during longer exposures probably due to the presence of a greater number of reactive species that can promote the formation of intermolecular reactions. Furthermore, the changes in the tan*δ* value, i.e., the value of *G*″/*G*′, as a function of angular frequency, are also shown in Figure 2. A value of tan*δ* < 1 indicates elastic-like behavior, while tan*δ* > 1 indicates viscous-like behavior [35]. Since the tan*δ* values of chia mucilage samples were lower than 0.6, it can be stated that mucilages behave more elastically than viscous materials, meaning that the deformation will mostly be elastic and recoverable [36]. However, higher tan*δ* values were obtained in the CP-treated mucilages compared to the control group. Therefore, it may be suggested that CP-treated mucilages could provide benefits in spreadable food formulations as they will be more cohesive and shear-resistant compared to control mucilage, which is less cohesive and brittle.

Power-law parameters calculated from the results of the frequency sweep test of mucilage samples are given in Table 1. Accordingly, all mucilage samples can be classified as physical gels since the *B* values are higher than 0. The *B* value indicates the strength and maturity of gels and is related to the frequency dependency of *G*′. The *A* value is also related to the elasticity of the structure [11,37]. The CP treatment of chia seeds has affected these parameters. Compared to the control group, CP treatment for 30 and 60 s significantly increased the *B* value (*p* < 0.05), while increasing the treatment time to 120 s decreased this value insignificantly (*p* > 0.05). Moreover, the increase in the *B* value of the CP30 and CP60 groups was accompanied by a decrease in the *A* value (*p* < 0.05), meaning that weaker and more frequency-dependent gels were obtained. Conversely, the *A* value of the CP120 group significantly increased (*p* < 0.05). Therefore, it can be stated that the longer exposures to CP were favorable in terms of gel maturity and strength. Similar results were also found in the work of Sadeghi et al. [33] for *L. perfoliatum* seed gum, where the *A* values increased and the *B* values decreased with CP treatment time at 5 kV voltage. However, Amirabadi et al. [15] reported that increasing the CP treatment duration of guar gum led to the weakening of the elastic properties. 

### 3.3. LAOS

**Figure 3:**
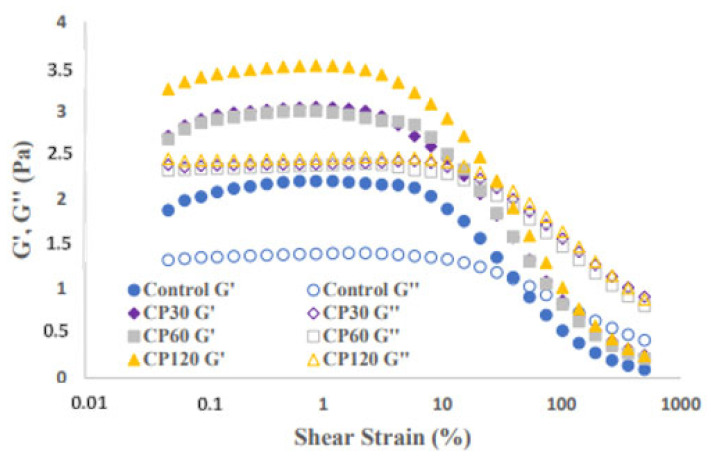
G′ (𝛾_0_) and G′′ (𝛾_0_) values of mucilage gel samples (1% *w*/*v*).

The effect of strain amplitude on *G*’ (*𝛾*_0_) and *G*” (*𝛾*_0_) of 1% (*w*/*v*) gels of mucilage samples is shown in Figure 3. As seen, the linear viscoelastic region (LVR) of the control, CP30, and CP60 groups continued up to 8.04% strain amplitude, while the LVR of CP120 ended at 11% strain amplitude. A longer LVR means a more elastic and flexible structural network formed by intermolecular forces, entanglements, and attractive forces between polysaccharide chains [38]. *G*′ values were higher than *G*” values in all mucilage samples. However, *G*′ values started to decrease and approached *G*” values as strain amplitude increased beyond the LVR, indicating the structure began to lose its energy storage ability [39]. According to the classification proposed by Hyun et al. [40], all samples showed Type I strain-thinning behavior, which is common for suspensions and biopolymer solutions. It is a result of the interaction of weak molecules and is generally observed in weakly-associated systems where polymer entanglements occur. The presence of large amplitudes causes deformations and destruction of the formed structure and reorientation of the aggregated particles into the flow direction. Moreover, the G′ values of CP-treated mucilages were higher than the control, with the highest values being recorded for the CP120 group. This means that a more stable structure was obtained with CP treatment for 120 s, probably due to the cross-linking effects [21], which is consistent with the small amplitude oscillatory shear results. 

**Figure 4:**
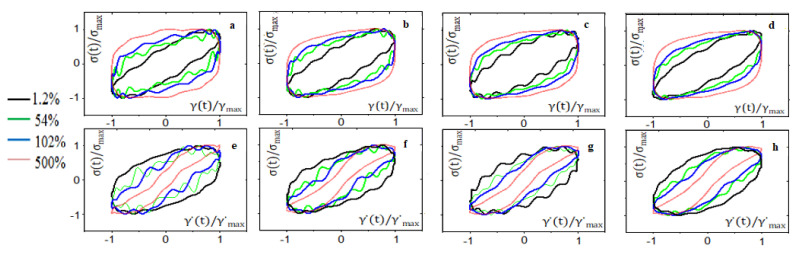
Normalized elastic (**a**–**d**) and viscous (**e**–**h**) Lissajous-Bowditch curves at 10 rad/s for chia mucilage gel samples; (**a**,**e**) belong to the control, (**b**,**f**) belong to CP30, (**c**,**g**) belong to CP60, and (**d**,**h**) belong to CP120.

The effect of CP treatment on the large deformation behavior of chia mucilages was summarized in the form of Lissajous plots. Figure 4a–d shows the normalized elastic Lissajous curves in order to visualize the differences in the nonlinear stress response of gels depending on the deformities in the elliptical shape of the curves with an increase in the strain amplitude. The alterations in the normalized stress responses (σ/σ_max_) versus the applied normalized strain (γ/γ_max_) were represented by black (1.2% strain), green (54% strain), blue (102% strain), and red (500% strain) lines. Accordingly, the elastic Lissajous curves were ellipsoidal at a small amplitude (black line), which shows that the response was dominated by elasticity. In the elastic perspective of elastic materials, the stress response to applied strain is reflected as a rectilinear or narrow elliptical shape at low strains, while the elliptical shapes become wider as the strain is increased [41]. As strain increased into the nonlinear regime, the shapes were deformed and nonlinearities were observed as distortions of the elliptical shape, indicating that the response was starting to be dominated by viscous behavior. These distortions were attributed to the different forms of microstructures, shear-induced structures, and responses when subjected to large deformations [42]. Lissajous-Bowditch curves changed from an ellipse to a rounded parallelogram as the strain amplitude increased, indicating increased viscous dissipation and a shift from elastic-dominated to viscous-dominated behavior. Although the structure of the material is completely recoverable following the deformation at small amplitudes in the linear region, it is likely that increasing the strain amplitude increased the amount of permanent deformation, thus changing the sample’s behavior to viscous-dominated behavior as it would not be able to elastically store as much stress energy due to network disruption [43]. Moreover, the elastic Lissajous curves of gels prepared from mucilages of CP-treated seeds, especially the CP120 sample, were smoother compared to the control mucilage, indicating that the CP treatment produced a more elastic network. Similar behavior was observed in the study of pectin solutions by Ozmen et al. [19]. The authors found that when the molecular weight of polyethylene oxide (PEO) and the concentration of pectin were increased, the elastic Lissajous curves were smooth even at high strains, and they attributed this finding to the increased number of physical bonds at higher pectin concentrations and molecular weights of PEO. Therefore, the smoother curves obtained for CP-treated samples can be explained by the fact that the CP treatment created a more cohesive network with more physical bonds, which requires more energy to destabilize the system. 

The viscous Lissajous curves showing normalized stress responses (σ/σ_max_) to the applied normalized strain rates ($\dot{\gamma }$/$\dot{\gamma }$_max_) are given in Figure 4e–h. The alterations in the normalized stress responses (σ/σ_max_) versus the applied normalized strain rates ($\dot{\gamma }$/$\dot{\gamma }$_max_) were represented by black (1.2% strain), green (54% strain), blue (102% strain), and red (500% strain) lines. In viscous Lissajous curves, the stress response to the applied strain rate is reflected as a straight line or a narrow ellipse for perfectly viscous materials, while it deviates from linear viscous behavior and becomes a larger ellipse or perfect circle as the materials show more elastic behavior [44]. In agreement with this, the viscous projections of the gel samples were circularly shaped at a small strain amplitude (1.2% strain), but they became narrower as the strain amplitude increased, demonstrating that the response was changed from elastically-dominated gel to viscously-dominated fluid. This behavior confirmed the case revealed by the elastic perspective and showed the breakdown of the gel network of the mucilages. Similar to the elastic perspective, a smoother curve was obtained in the CP120 group compared to other groups. 

### 3.4. Color Analysis

The color properties of mucilage samples obtained from the chia seeds are shown in Table 2. Accordingly, CP treatment of chia seeds has significantly affected all the color parameters of the mucilages obtained (*p* < 0.05). It was found that the lightness (L*) values of the CP-treated mucilages decreased, while the a* and b* values increased in a time-dependent manner. Hussain et al. [22] reported that, since the seeds were exposed to high temperatures (up to 80 °C) during the hot extraction of mucilage, browning reactions could occur and negatively affect the color properties of mucilages. However, in this study, the mucilages were obtained by the cold extraction method, and thus, the hypothesis of browning reactions that could occur during extraction and drying was excluded. Therefore, the changes in the color properties can be attributed to the reactive plasma species that contact chia seeds during CP treatment. Reactive plasma species, such as H^+^, H_3_O^+^, O^+^, OH^−^, etc., may induce different chemical reactions [45]. For example, they can unfold the proteins and increase the contact area between protein and sugar, exposing more sites and providing adequate energy for Maillard reactions [46,47]. The plasma jet configuration used for the treatment of chia seeds can increase the substrate temperature up to 60–70 °C even for short processing times (<120 s), providing favorable conditions for the Maillard reactions. Moreover, the reactive plasma species can affect enzyme activity, such as polyphenol oxidase (PPO). According to Dantas et al. [48], the increase in the PPO activity of CP-treated açai pulp was accompanied by decreased L* values and increased b* values. In addition, the total color difference (∆E) values of chia seeds increased with the CP treatment time. In general, an ∆E value of 1.5–3.0 identifies a color difference that is barely visible to the naked eye [49]. Accordingly, it can be accepted that the treatment for 30 s did not induce a visible color difference. However, the color difference was noticeable with the naked eye for the CP60 and CP120 groups.

### 3.5. FTIR Analysis

**Figure 5:**
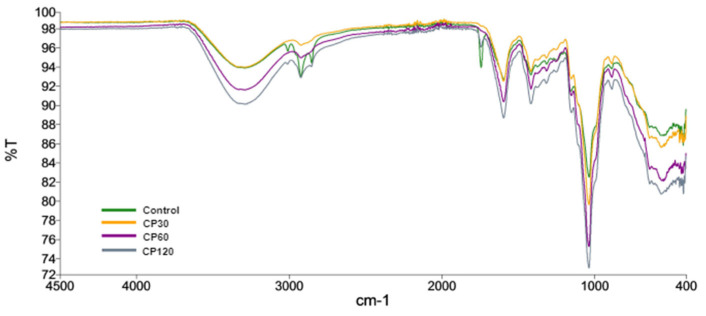
FTIR spectra of mucilage samples extracted from untreated (control) and CP-treated chia seeds.

The effect of CP treatment of chia seeds on the FTIR spectrum of extracted mucilage samples is presented in Figure 5. Accordingly, all samples showed a similar FTIR spectrum with some minor differences, which could be attributed to the effect of CP and differences in the proximate composition. A wide absorption band was observed at ~3290 cm^−1^, which can be assigned to the -OH groups [50]. CP treatment for 30 s did not affect this band, while longer treatments have increased the absorbance peak of -OH groups. Sadeghi et al. [33] found that the absorbance peak of the -OH groups of *L. perfoliatum* seed gum increased after low-voltage CP (5 kV) treatment, while it decreased after high-voltage CP (10 kV) treatment. The authors attributed these changes to the cross-linking and depolymerization effects of CP, respectively. However, in the case of chia mucilage, since the gas used to produce CP was argon, which is generally used for cross-linking [51], and the treatment times were relatively short, the increase in the absorbance peak of -OH groups may be attributed to the incorporation of -OH groups on the surface during exposure rather than the depolymerization effect [52]. The peaks at ~2924 cm^−1^ and ~2852 cm^−1^ are attributed to the C-H stretching in methyl and methylene groups [53]. The relative intensities and positions of these peaks did not change with CP treatment. The bands at ~1597 cm^−1^ and ~1417 cm^−1^ are related to the carboxyl groups of uronic acid or the presence of proteins in the mucilages [54]. The peak at ~1038 cm^−1^ is due to the C-O-C of 1–4 glycosidic bond ring vibrations, and it seems that CP treatment enhanced the intensity of this peak in a treatment time-dependent manner [53]. This may be attributed to the cross-linking effect of argon CP treatment. Moreover, the CP treatment did not induce the formation of a new absorbance peak at 1730 cm^−1^, which might be attributed to the carboxylic acid, meaning that the oxidation of chia mucilage was not induced [13]. Peaks between 700 and 500 cm^−1^ are associated with the crystallinity of chia gum, and they seem to be unaffected by CP treatment [55].

### 3.6. SEM Results

**Figure 6:**
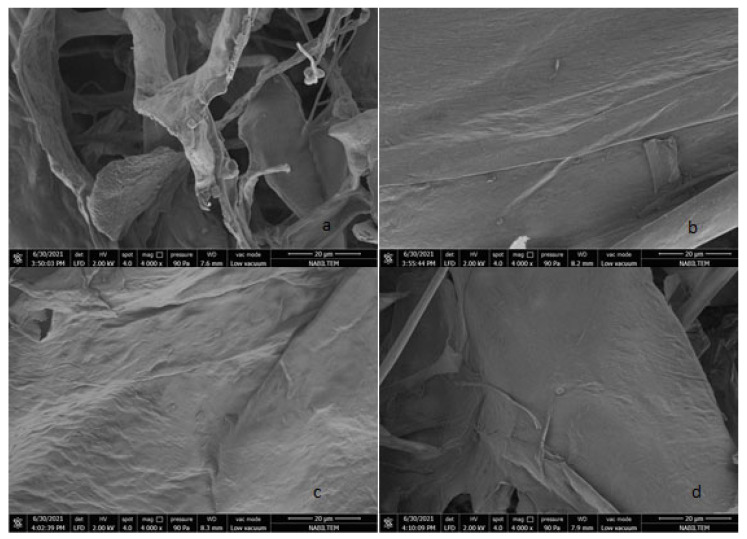
SEM micrographs of chia mucilages at 4000× magnification. (**a**) Control, (**b**) CP30, (**c**) CP60, and (**d**) CP120.

Figure 6 shows the SEM micrographs of freeze-dried mucilages obtained from chia seeds. Similar to freeze-dried chia mucilages obtained in the work of Tavares et al. [7], the porous structure was clearly seen in the control group. Capitani et al. [56] investigated the microscopic properties of fresh and freeze-dried chia mucilage using SEM and found that the association between different components that formed the mucilage forms a structure of open pores, providing different rheological properties to mucilage. The typical fiber strands of chia mucilage become overlapping sheets when the mucilage is freeze-dried [28,56]. The drying method of chia mucilage significantly affects its morphology and, thus, its functional and rheological properties. For example, hot air drying induces the formation of laminar plate aggregates with brittle aspects and less uniformity compared to their freeze-dried counterparts due to the thermal degradation during high-temperature drying [7]. Treatment for 30 s seems to produce a smooth sheet structure, probably due to the promotion of molecular interactions. Increasing the treatment time to 60 s induced more etching on the surface of the mucilage, causing a rougher structure. Sadeghi et al. [33] reported that, compared to the smooth surface of natural *L. perfoliatum* seed gum, the CP-treated gum surface had pinholes, fissures, scratches, and tiny particles due to the etching phenomena. For the CP120 group, a denser aggregate structure was obtained. Similarly, Silva et al. [28] found that the application of ultrasound to chia mucilage led to denser aggregates, but increasing the treatment time resulted in a better separation of the fibrils from the aggregates. Capitani et al. [56] reported that the clear strand structure becomes denser when the mucilage concentration is increased. Therefore, the denser structures obtained in the CP-treated samples are probably a result of increased intermolecular interactions and are responsible for the improvements in the rheological properties of mucilage. 

## 4. Conclusions

In this work, CP with argon as a working gas was used in the modification of chia mucilage. Overall, the treatment of chia seeds with CP at different times changed the rheological properties of the freeze-dried mucilages. The gels prepared from the mucilages of CP-treated chia seeds showed higher viscosity than that of the control group over the applied shear range, with the highest values being recorded for the CP120 group. A frequency sweep test revealed that chia mucilages are weak gels, and CP treatment produced a more stable network depending on treatment time. LAOS analysis provided valuable information about the effect of CP treatment on the large deformation behavior of chia mucilages. It was found that the CP treatment has affected the LAOS behavior and increased the deformation resistance of mucilages. All mucilage gels showed elastic and Type I strain-thinning behavior. The color parameters of mucilages were affected by CP treatment, and the L* value showed a decreasing trend while the a* and b* values showed an increasing trend with treatment time. FTIR analysis revealed the incorporation of -OH groups on the surface and the formation of a more stable structure. CP treatment produced denser structures, as visualized by SEM. In conclusion, it seems that CP treatment is an effective way to modify both the SAOS and LAOS properties of food gums.

## Figures and Tables

**Table 1:** Herschel-Bulkley parameters for the relationship between shear stress and shear rate and Power-law parameters for the relationship between storage modulus and frequency of mucilage gel samples (1% *w*/*v*).

Sample	Steady Flow Test				Dynamic Viscoelastic Test		
	*K* (Pa s^n^)	*n* (-)	*σ*_0_	*R* ^2^	*A* (Pa s)	*B* (-)	*R* ^2^
Control	0.769 ^c^	0.598 ^a^	2.440 ^a^	0.996	5.628 ^b^	0.207 ^b^	0.9072
CP30	1.199 ^b^	0.452 ^c^	1.606 ^b^	0.989	3.790 ^c^	0.239 ^a^	0.9816
CP60	1.255 ^b^	0.566 ^ab^	1.446 ^b^	0.998	4.695 ^bc^	0.261 ^a^	0.9431
CP120	1.928 ^a^	0.532 ^b^	2.694 ^a^	0.996	9.563 ^a^	0.196 ^b^	0.9516

*K* is the consistency coefficient, *n* is the flow behavior index, *σ*_0_ is the yield stress (Pa), and *A* and *B* are the constants of the Power-law parameters for the relationship between storage modulus and frequency. Different lower-case letters indicate a statically significant difference among different treatments.

**Table 2:** Color analysis results of mucilage samples.

Sample	L*	a*	b*	∆E
Control	89.89 ± 0.07 ^a^	0.17 ± 0.00 ^d^	0.74 ± 0.01 ^d^	-
CP30	86.97 ± 0.02 ^b^	0.37 ± 0.01 ^c^	1.23 ± 0.00 ^c^	2.96
CP60	81.30 ± 0.02 ^c^	1.23 ± 0.01 ^b^	3.39 ± 0.01 ^b^	9.05
CP120	76.16 ± 0.01 ^d^	1.83 ± 0.03 ^a^	5.02 ± 0.00 ^a^	14.47

The data represent the average values ± standard deviation of three independent samples. There is no statistical difference between the results shown with the same exponential lowercase letter in the same column (*p* > 0.05). CP: Cold atmospheric pressure plasma jet. CP30, CP60, CP120: CP treatment times (s).

## Data Availability

Data available on request due to ethical restrictions.

## Supplementary Materials

The following supporting information can be downloaded at: https://www.mdpi.com/article/10.3390/foods12081563/s1, Figure S1: Flow curve data of 1% (*w*/*v*) gels of mucilage samples from untreated (control) and CP-treated chia seeds for Power-law and Casson rheological models.
