# Supplementary material for: Effect of Cold Atmospheric Pressure Argon Plasma Jet Treatment on the Freeze-Dried Mucilage of Chia Seeds (Salvia hispanica L.)
Source: Foods. 2023 Apr 7;12(8):1563. doi: 10.3390/foods12081563 (PMC10137730; doi:10.3390/foods12081563)
Supplement: Supplementary file 1 [file foods-12-01563-s001.zip › foods-2277464-supplementary.pdf]

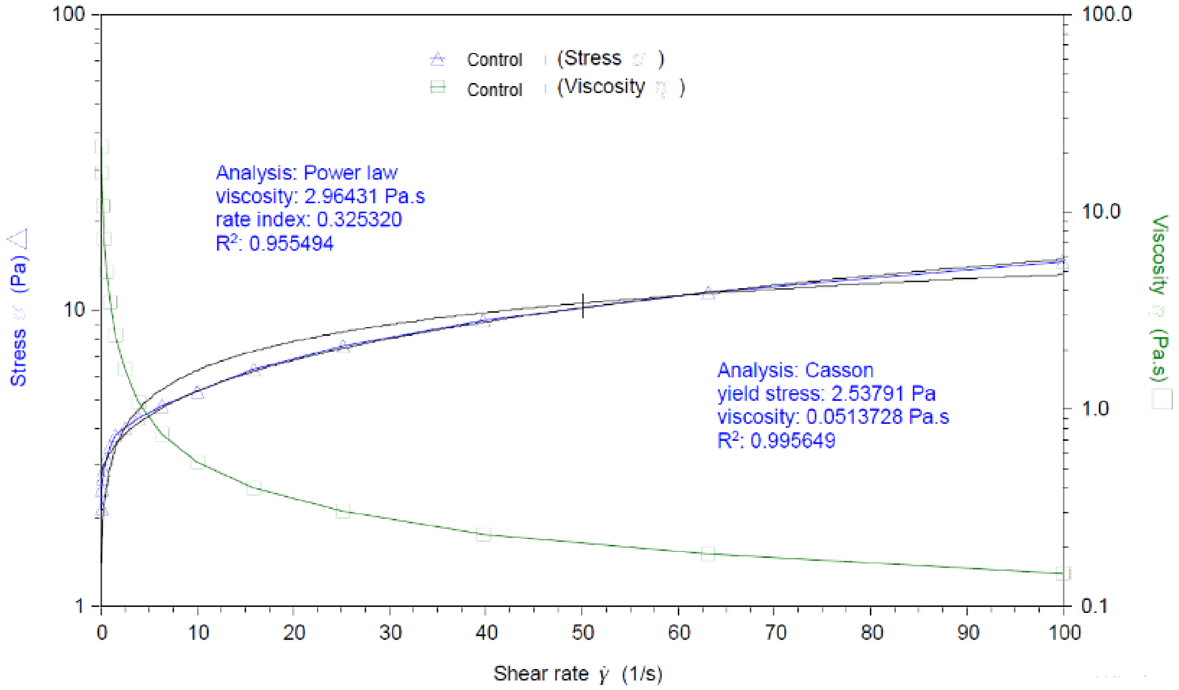

Control (Stress σ)
Control (Viscosity η)
Analysis: Power law
viscosity: 2.96431 Pa.s
rate index: 0.325320
R²: 0.955494
Analysis: Casson
yield stress: 2.53791 Pa
viscosity: 0.0513728 Pa.s
R²: 0.995649
Stress σ (Pa)
Viscosity η (Pa.s)
Shear rate γ̇ (1/s)

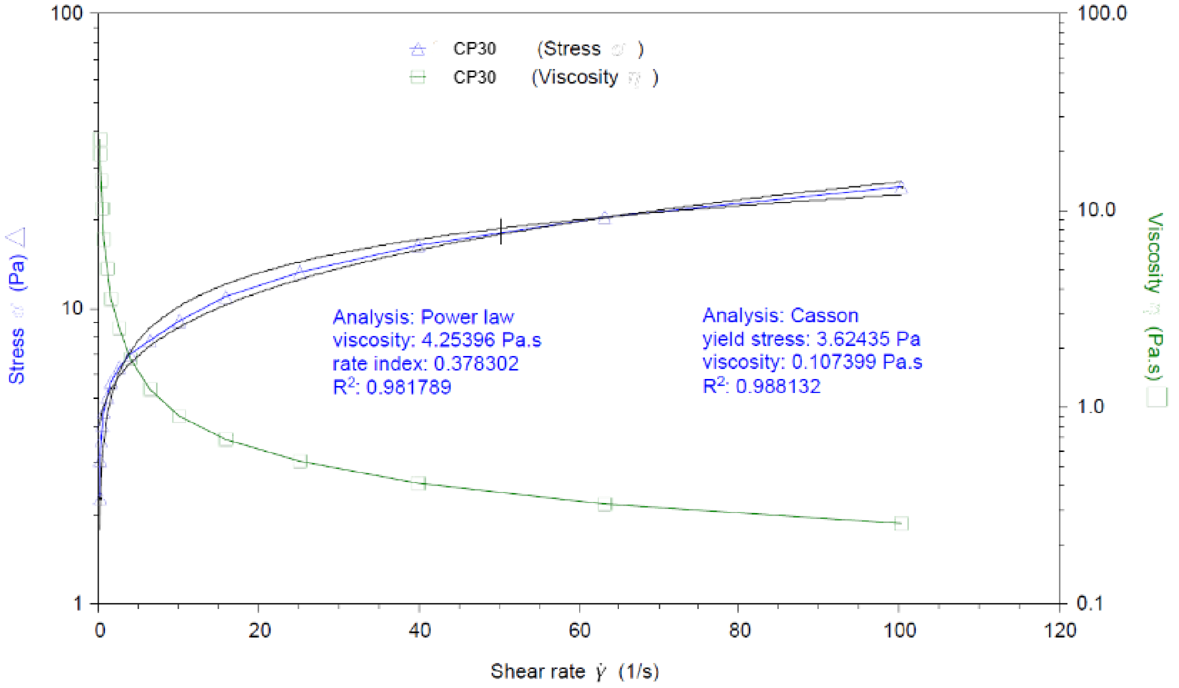

CP30 (Stress σ)
CP30 (Viscosity η)
Analysis: Power law
viscosity: 4.25396 Pa.s
rate index: 0.378302
R²: 0.981789
Analysis: Casson
yield stress: 3.62435 Pa
viscosity: 0.107399 Pa.s
R²: 0.988132
Stress σ (Pa)
Viscosity η (Pa.s)
Shear rate γ̇ (1/s)

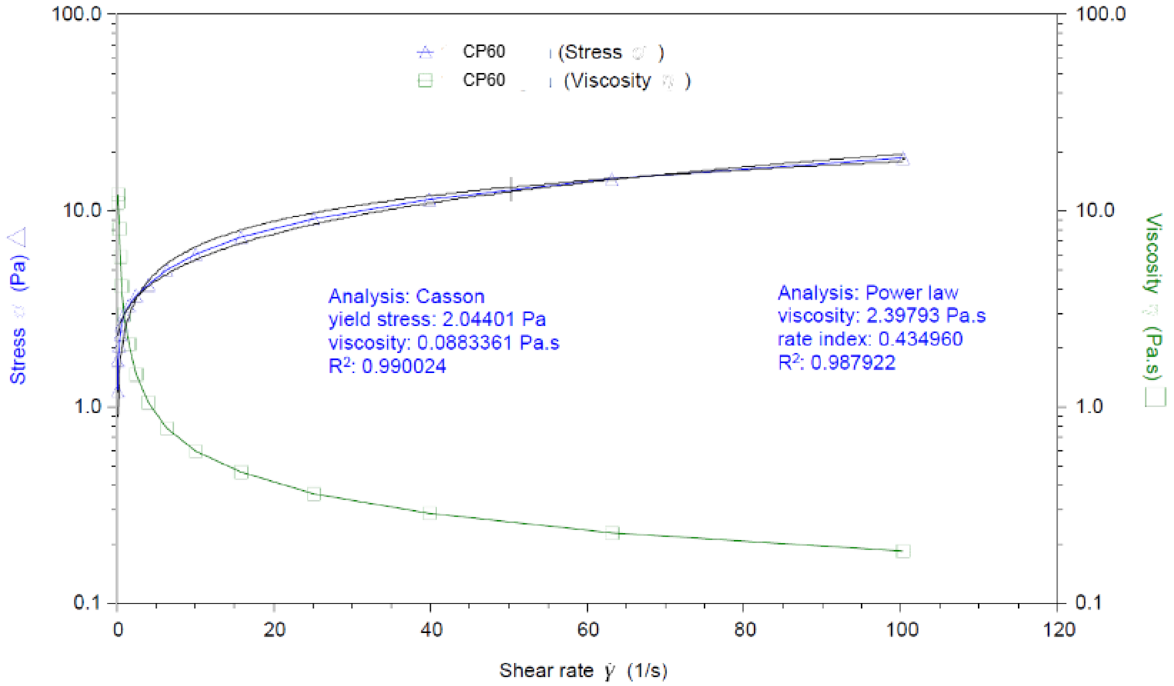

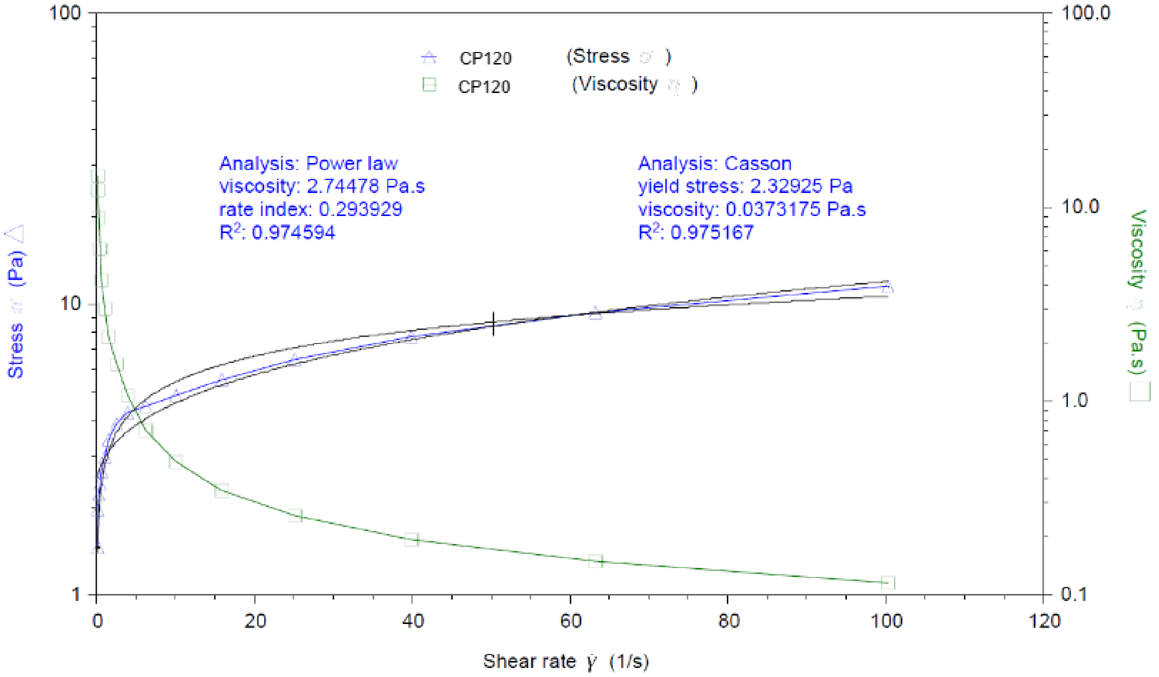

Figure S1. Flow curve data of 1% (w/v) gels of mucilage samples from untreated (control) and CP-treated chia seeds for Power-law and Casson rheological models.
